# OLDER PARENTS’ PERCEIVED SOCIAL SUPPORT AND STRAIN FROM ADULT CHILDREN AND INTERGENERATIONAL FINANCIAL TRANSFER

**DOI:** 10.1093/geroni/igz038.1035

**Published:** 2019-11-08

**Authors:** Dahee Kim, Peter Martin

**Affiliations:** Iowa State University, Ames, Iowa, United States

## Abstract

The gerontological literature indicates that both positive and negative relationships are dimensions of intergenerational relationships. Moreover, depending on intergenerational financial support, the association between older parents’ perceived social support and strain from adult children can vary. The purpose of the current study is to investigate the association between intergenerational social support and strain. We also examined the impact of intergenerational financial transfers on intergenerational social support and strain. We analyzed data of 1,329 older adults aged 65 to 84 from the Health and Retirement Study collected at 2006(t1), 2010(t2), and 2014(t3). Cross-lagged panel models were performed to examine the reciprocal association between intergenerational social support and strain over time. Multiple group comparisons were conducted to estimate the impact of financial support exchange on social support and strain from adult children. The results demonstrated that social support and strain from adult children were stable over time. Furthermore, social strain had negative effects on the changes in social support from adult children. Multiple group comparisons suggested that in the parents’ groups (financial support provision vs. no provision to adult children, and financial support receipt vs. no receipt groups from adult children) intergenerational social support and strain were stable over time. Additionally, the impact of social strain on subsequent social support from adult children differed depending on intergenerational financial support. These findings highlight the reciprocal association between intergenerational positive and negative relationships. Further, this research suggests the importance of intergenerational support in older parents’ and adult children’s positive and negative relationship quality.

